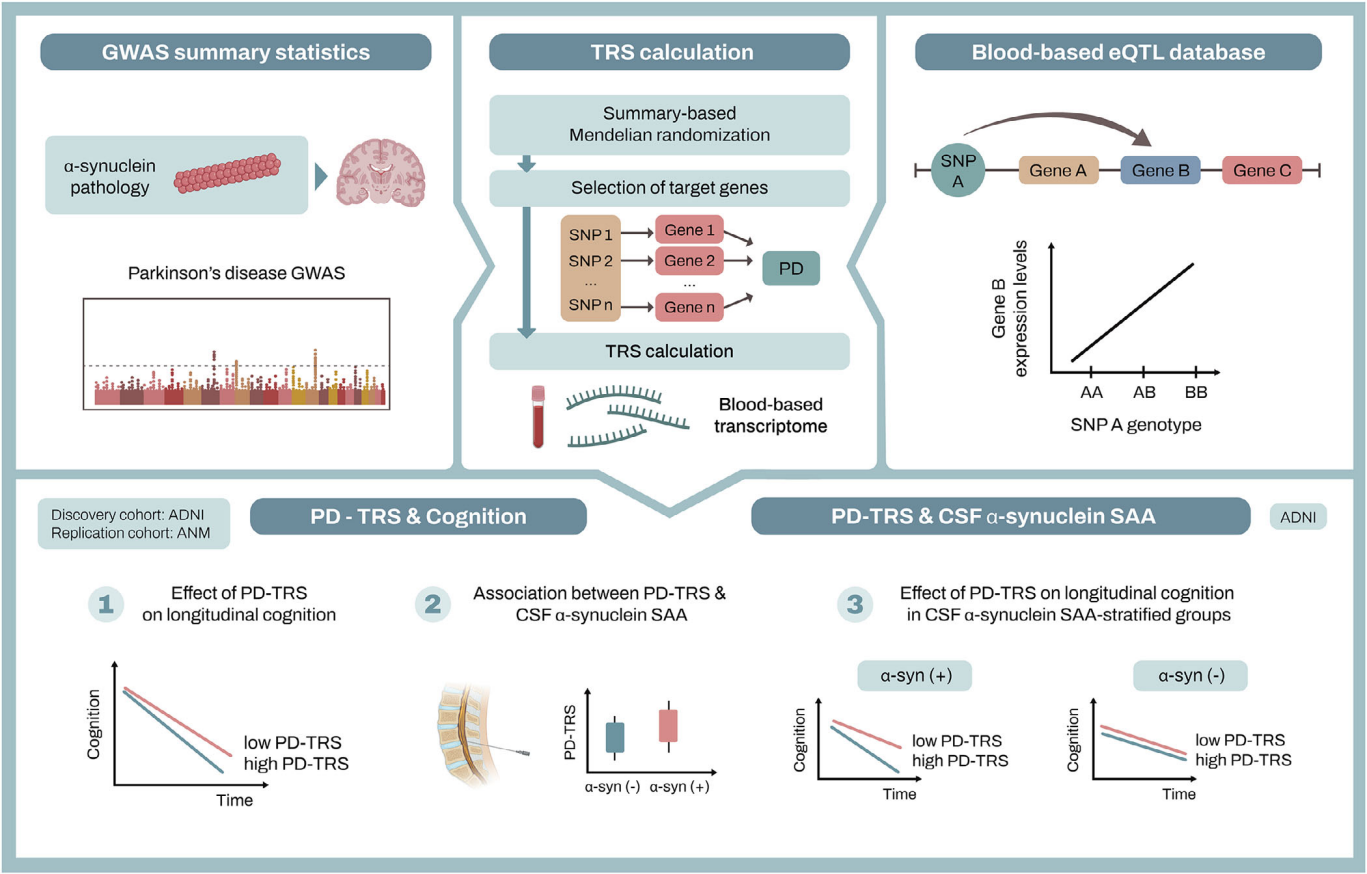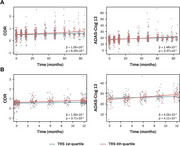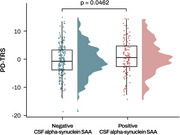# Blood‐based transcriptional risk score for Parkinson's disease is associated with cognitive decline in Alzheimer's disease

**DOI:** 10.1002/alz70856_099214

**Published:** 2025-12-25

**Authors:** Jung‐Min Pyun, Young Ho Park, Duygu Tosun, Paula J Bice, SangYun Kim, Michael S. W. Weiner, Andrew J. Saykin, Kwangsik Nho

**Affiliations:** ^1^ Soonchunhyang University Seoul Hospital, Seoul, Korea, Republic of (South); ^2^ Seoul National University Bundang Hospital, Seongnam, Korea, Republic of (South); ^3^ Department of Veterans Affairs Medical Center, Northern California Institute for Research and Education (NCIRE), San Francisco, CA, USA; ^4^ Department of Radiology and Biomedical Imaging, University of California, San Francisco, San Francisco, CA, USA; ^5^ Indiana University School of Medicine, Indianapolis, IN, USA; ^6^ Seoul National University Bundang Hospital, Seoul National University College of Medicine, Seongnam, Gyeonggi‐do, Korea, Republic of (South); ^7^ University of California, San Francisco, San Francisco, CA, USA; ^8^ Northern California Institute for Research and Education (NCIRE), San Francisco, CA, USA; ^9^ Department of Radiology and Imaging Sciences, Center for Neuroimaging, School of Medicine, Indiana University, Indianapolis, IN, USA; ^10^ Department of Radiology and Imaging Sciences, Indiana Alzheimer's Disease Research Center, Center for Neuroimaging, Indiana University School of Medicine, Indianapolis, IN, USA

## Abstract

**Background:**

Mixed pathology in Alzheimer's disease (AD) contributes to diverse endophenotypes and disease progression. While alpha‐synucleinopathy often co‐occurs with AD, its transcriptional risk effect on clinical outcomes is not well understood. We developed a blood‐based transcriptional risk score for Parkinson's disease (PD‐TRS), a neurodegenerative disease resulting from alpha‐synucleinopathy.

**Method:**

We prioritized target genes by integrating PD genome‐wide association study summary and expression quantitative trait locus data using Mendelian randomization and calculated PD‐TRS using blood transcriptome data in two independent cohorts: Alzheimer's Disease Neuroimaging Initiative (ADNI, n = 661) and ANMerge (ANM, n = 665). We performed association analysis of PD‐TRS with longitudinal cognitive function. Additionally, we investigated the association between PD‐TRS and the presence of alpha‐synuclein seeds in cerebrospinal fluid (CSF) detected using a seed amplification assay (SAA), and the effect of PD‐TRS on longitudinal cognition in CSF alpha‐synuclein SAA‐stratified groups (Figure 1).

**Result:**

Our analysis revealed that higher PD‐TRS was associated with rapid cognitive decline rates in both cohorts (Figure 2). Furthermore, higher PD‐TRS was significantly associated with the CSF alpha‐synuclein SAA positivity in the ADNI (Figure 3). We also found a significant interaction effect of PD‐TRS and the CSF SAA positivity on longitudinal cognitive changes. Only in the CSF SAA‐positive group, higher PD‐TRS was associated with faster cognitive decline.

**Conclusion:**

Our findings suggest that blood‐based PD‐TRS may reflect the CSF alpha‐synuclein SAA positivity and longitudinal cognitive decline in comorbid alpha‐synucleinopathy in AD, advancing the identification of a blood‐based biomarker to assess mixed pathology in AD.